# Fine Particulate Matter Exposure From Secondhand Cannabis Bong Smoking

**DOI:** 10.1001/jamanetworkopen.2022.4744

**Published:** 2022-03-30

**Authors:** Patton Khuu Nguyen, S. Katharine Hammond

**Affiliations:** 1 Environmental Health Sciences Division, School of Public Health, University of California, Berkeley

## Abstract

This cohort study quantifies the levels of secondhand cannabis smoke from social bong smoking in a home environment.

## Introduction

Secondhand cannabis smoke (SHCS) is a novel exposure source uncharacterized in homes but containing known health risk factors.^1^ Although 27% of young adults believe SHCS exposure is safe,^2^ cannabis smoke has several hundred toxic chemicals, carcinogens, and fine particulate matter (PM_2.5_), many at higher concentrations than tobacco smoke.^1^ Decades of secondhand tobacco smoke (SHTS) research demonstrate causal links to cancer, respiratory and cardiovascular diseases, preterm birth, and decreased immune function.^3^ These concerns have not translated to cannabis bong smoking, a popular consumption method in social settings among young adults, wherein smoke is drawn through water. However, like SHTS, 1 minute of SHCS caused significant endothelial dysfunction in rats.^4^ This cohort study measured PM_2.5_ levels from social bong smoking; it is the first, to our knowledge, to quantify SHCS levels from social cannabis smoking in the home.

## Methods

Levels of PM_2.5_ were measured before, during, and after 8 cannabis social-smoking sessions in one 20-m^2^ household living room (eMethods in the Supplement). An aerosol monitor (SidePak AM510; TSI Inc) measured PM_2.5_ concentrations where a nonsmoker might sit. The University of California, Berkeley, Office for the Protection of Human Subjects deemed this study not human participants research and waived review. This study followed the STROBE reporting guideline. The Wilcoxon rank sum 2-sided test assessed statistical differences between PM_2.5_ concentrations before and during smoking. Analysis was performed using RStudio, version 1.4 (RStudio). Two-sided *P* < .05 indicated statistical significance.

## Results

Home cannabis bong smoking significantly increased PM_2.5_ from background levels (conditions existing before the smoking began) in all sessions by 100-fold to 1000-fold for 6 of 8 sessions; the other 2 sessions had high background and significantly increased PM_2.5_ more than 20-fold (*P* < .001 for all 8 sessions). During the first 10 minutes of smoking, mean (SD) PM_2.5_ concentrations increased to 410 (220) μg/m^3^, after 15 minutes to 570 (290) μg/m^3^, after 30 minutes to 1000 (320) μg/m^3^, and went as high as 2500 μg/m^3^ in 1 session (Figure). The concentration during smoking increased to a mean (SD) of 1300 (280) μg/m^3^ (Table). During 2-hour smoking sessions, mean (SD) 5-minute peak PM_2.5_ concentration was 1700 (460) μg/m^3^ and remained half that 90 minutes after smoking ceased. Each half hour after smoking ceased, mean concentration declined to 78% of peak value, then 60%, then 40%, and, after 110 minutes, 31%. In the 1 session monitored for 12 hours after smoking stopped, PM_2.5_ remained elevated at 50 μg/m^3^, more than 10 times the background concentration. Cannabis bong smoking in the home generated 4 times greater PM_2.5_ concentrations than cigarette or tobacco hookah (waterpipe) smoking (Table).

**Figure.**
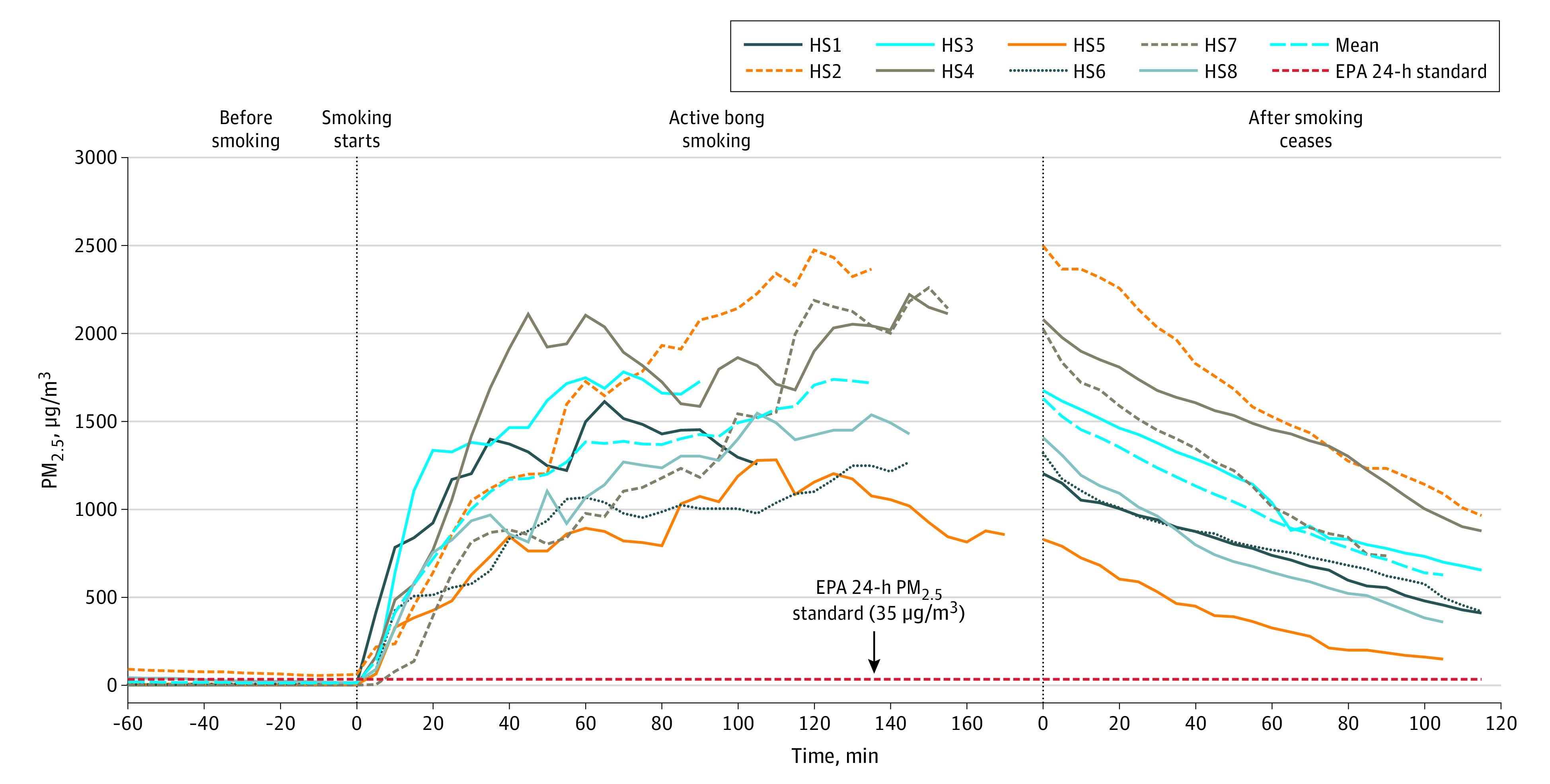
Continuous Fine Particulate Matter (PM_2.5_) Concentrations During 8 Household Smoking (HS) Sessions When Doors Were Closed Each HS session is delineated by a colored line. The dashed blue line represents the mean concentration. Active smoking lasted 90 to 170 minutes. The dashed red line denotes the US Environmental Protection Agency (EPA) national ambient air quality standard for 98th percentile 24-hour PM_2.5_ exposure (35 μg/m^3^).

**Table.** PM_2.5_ Secondhand Cannabis and Tobacco Smoke Exposure Concentrations in Homes^a^

Smoke source	No. of experiments^b^	Sampling		PM_2.5_ concentration, mean (SD), μg/m^3^	Source
		Method^c^	Time, min		
Cannabis bong	8	SidePak	140	1300 (280)	This study
Tobacco					
Hookah (waterpipe)	12	DataRAM	42	310 (620)	Shearston et al,^5^ 2021
Cigarette	15	DataRAM	23	330 (400)	Shearston et al,^5^ 2021
	7	SidePak	40	320 (190)	Acevedo-Bolton et al,^6^ 2014

Abbreviation: PM_2.5_, fine particulate matter with an aerodynamic diameter up to 2.5 μm.

^a^ Measurement of PM_2.5_ exposure concentrations from ad libitum cannabis or tobacco smoking in homes located in the US. Home smoking and sampling in each study occurred in similar living room settings.

^b^ Number of smoking sessions in which PM_2.5_ concentration was measured.

^c^ Personal aerosol monitors measured PM_2.5_ concentrations: SidePak AM510 (TSI Inc) and DataRAM (Thermo Scientific).

## Discussion

The PM_2.5_ concentrations generated in a home during social cannabis bong smoking to which a nonsmoking resident might be exposed were greatly increased compared with background levels, and PM_2.5_ decayed only gradually after smoking ceased. After 15 minutes of smoking, mean PM_2.5_ (570 μg/m^3^) (Figure) was more than twice the US Environmental Protection Agency (EPA) hazardous air quality threshold (>250 μg/m^3^). If one assumes the exposure concentrations were at the mean levels observed, a single home smoking session with no other exposures would generate an estimated mean daily concentration (200 μg/m^3^) that greatly exceeds the average in cigarette-smoking homes (44 μg/m^3^), nonsmoking homes (15 μg/m^3^), and the US EPA daily standard (35 μg/m^3^).^3^ A strength of this study is that measurements were made during actual social bong smoking sessions without artificial constraints. Limitations include that cannabis smoking was not directly observed.

This cohort study suggests that, contrary to popular beliefs, bong smoking is not safe. Decades ago, many people thought SHTS presented no health risk to nonsmokers. Scientific research since then changed this perception and led to smoke-free environments.^3^ Incorrect beliefs about SHCS safety promote indoor cannabis smoking.^1,2^ Nonsmokers are exposed to even higher concentrations of SHCS materials during “hot-boxing,” the popular practice in which cannabis smokers produce high volumes of smoke in an enclosed environment. This study’s findings suggest SHCS in the home is not safe and that public perceptions of SHCS safety must be addressed.

## Notes

Supplement. **eMethods.** Supplemental Methods for Measuring Secondhand Cannabis Smoke
eReference
