# Supplementary material for: Fine Particulate Matter Exposure From Secondhand Cannabis Bong Smoking
Source: JAMA Netw Open. 2022 Mar 30;5(3):e224744. doi: 10.1001/jamanetworkopen.2022.4744 (PMC8968464; doi:10.1001/jamanetworkopen.2022.4744)
Supplement: Supplement. — eMethods. Supplemental Methods for Measuring Secondhand Cannabis Smoke eReference [file jamanetwopen-e224744-s001.pdf]

Supplemental Online Content

Nguyen PK, Hammond SK. Fine particulate matter exposure from secondhand cannabis bong smoking. *JAMA Netw Open*. 2022;5(3):e224744. doi:10.1001/jamanetworkopen.2022.4744

eMethods. Supplemental Methods for Measuring Secondhand Cannabis Smoke

eReference

This supplemental material has been provided by the authors to give readers additional information about their work.

© 2022 Nguyen PK et al. *JAMA Network Open.*

eMethods. Supplemental Methods for Measuring Secondhand Cannabis Smoke

Home and smoking conditions.

This study measured SHCS generated by cannabis bong smoking under real-life smoking conditions. Several smokers smoked at their own volition, ad libitum, in a social setting of their own choosing, not an experimental setting, and without any instructions or limitations from the investigators. Cannabis smoke was emitted from a 0.4 m tall Showerhead Breaker Bong (Diamond Glass) where 5-8 bowls were smoked per session. All samples and observations were collected without interacting with the smokers. The SidePak AM510, which measured $PM_{2.5}$, was 0.86 m above the floor and 2.0 m from the bong. The location of the SidePak AM510 remained constant to simulate a seated bystander's potential exposure in the primary area in which the eight smoking sessions occurred. Smoking was not kept constant, nor directed in any way by the investigators. All windows and doors were observed to be closed for each session. Background concentrations were measured for 60 minutes before smoking began. Active smoking duration averaged 140 minutes. After smoking ceased, measurements continued for 90 to 180 minutes, except that in one session measurements continued for 12 hours after smoking ceased.

Gravimetric Analysis.

Light scattering detectors such as the SidePak AM510 must be calibrated for each type of PM being measured as their response depends both on the composition and the size distribution of the particles. Two $PM_{2.5}$ impactors were used to conduct gravimetric total mass sampling of PM during active smoking at 3 L/min. Each impactor was attached to a GilAir-5 pump that was calibrated before and after each social event. PTFE membrane plastic ring 37 mm filters were weighed, on a Mettler Toledo XP2U Cahn Balance, pre- and post- sampling after equilibration for 24 hours, to calculate total mass of SHCS collected during bong smoking sessions. PM concentrations during two sampling sessions were measured, and the resulting mass concentrations were used to calculate a gravimetric correction factor of 0.27 µg PM (gravimetric)/µg PM (reported by SidePak AM510). The calibration correction factor for cannabis bong smoking is comparable to cigarette smoking (0.24-0.32)[1].

Daily Concentration Analysis.

As an example, the daily concentration is calculated as a time weighted average, i.e., by multiplying the average PM concentration during smoking ($C_{during\ smoking}$) by the average smoking duration ($t_{duration\ smoking}$), then adding the products of average concentration (C_i) during each half hour of decay by 0.5 hours (t_i) for the first two hours, and dividing this sum by 24 hours. For this purpose, no other exposure was presumed to occur for the remaining 20 hours. The resulting concentration represents a potential daily PM exposure from social bong smoking.

$$Daily\ TWA = \frac{C_{during\ smoking} * t_{duration\ smoking} + \sum C_i t_i}{24\ hours}$$

Human Subjects Exemption.

As per the University of California, Berkeley, Office for the Protection of Human Subjects, this project is not human subjects research and does not require IRB review because this project was limited to collecting air samples within a home and data on the space itself (e.g., open windows, furnace, etc.), and not on the smokers.

© 2022 Nguyen PK et al. *JAMA Network Open.*

eReference

1. Acevedo-Bolton V, Ott WR, Cheng KC, Jiang RT, Klepeis NE, Hildemann LM. Controlled experiments measuring personal exposure to PM2.5 in close proximity to cigarette smoking. *Indoor Air*. 2014;24(2):199-212. doi:10.1111/ina.12057

© 2022 Nguyen PK et al. *JAMA Network Open.*
